# Discovery of NRG1-VII: the myeloid-derived class of NRG1

**DOI:** 10.1186/s12864-024-10723-2

**Published:** 2024-08-29

**Authors:** Miguel A Berrocal-Rubio, Yair David Joseph Pawer, Marija Dinevska, Ricardo De Paoli-Iseppi, Samuel S. Widodo, Josie Gleeson, Nadia Rajab, Will De Nardo, Jeannette Hallab, Anran Li, Theo Mantamadiotis, Michael B. Clark, Christine A. Wells

**Affiliations:** 1 Department of Anatomy and Physiology, Faculty of Medicine, Dentistry and Health Sciences, The University of Melbourne, Melbourne, Australia; 2 Department of Surgery, Royal Melbourne Hospital, The University of Melbourne, Melbourne, Australia; 3 Department of Microbiology and Immunology, Faculty of Medicine, Dentistry and Health Sciences, The University of Melbourne, Melbourne, Australia

**Keywords:** NRG1, Myeloid, Growth factor, Isoforms, Macrophages

## Abstract

The growth factor Neuregulin-1 (NRG1) has pleiotropic roles in proliferation and differentiation of the stem cell niche in different tissues. It has been implicated in gut, brain and muscle development and repair. Six isoform classes of NRG1 and over 28 protein isoforms have been previously described. Here we report a new class of NRG1, designated NRG1-VII to denote that these NRG1 isoforms arise from a myeloid-specific transcriptional start site (TSS) previously uncharacterized. Long-read sequencing was used to identify eight high-confidence NRG1-VII transcripts. These transcripts presented major structural differences from one another, through the use of cassette exons and alternative stop codons. Expression of NRG1-VII was confirmed in primary human monocytes and tissue resident macrophages and induced pluripotent stem cell-derived macrophages (iPSC-derived macrophages). Isoform switching via cassette exon usage and alternate polyadenylation was apparent during monocyte maturation and macrophage differentiation. NRG1-VII is the major class expressed by the myeloid lineage, including tissue-resident macrophages. Analysis of public gene expression data indicates that monocytes and macrophages are a primary source of NRG1. The size and structure of class VII isoforms suggests that they may be more diffusible through tissues than other NRG1 classes. However, the specific roles of class VII variants in tissue homeostasis and repair have not yet been determined.

**Supplementary Information:**

The online version contains supplementary material available at 10.1186/s12864-024-10723-2.

## Introduction

**Fig. 1:**
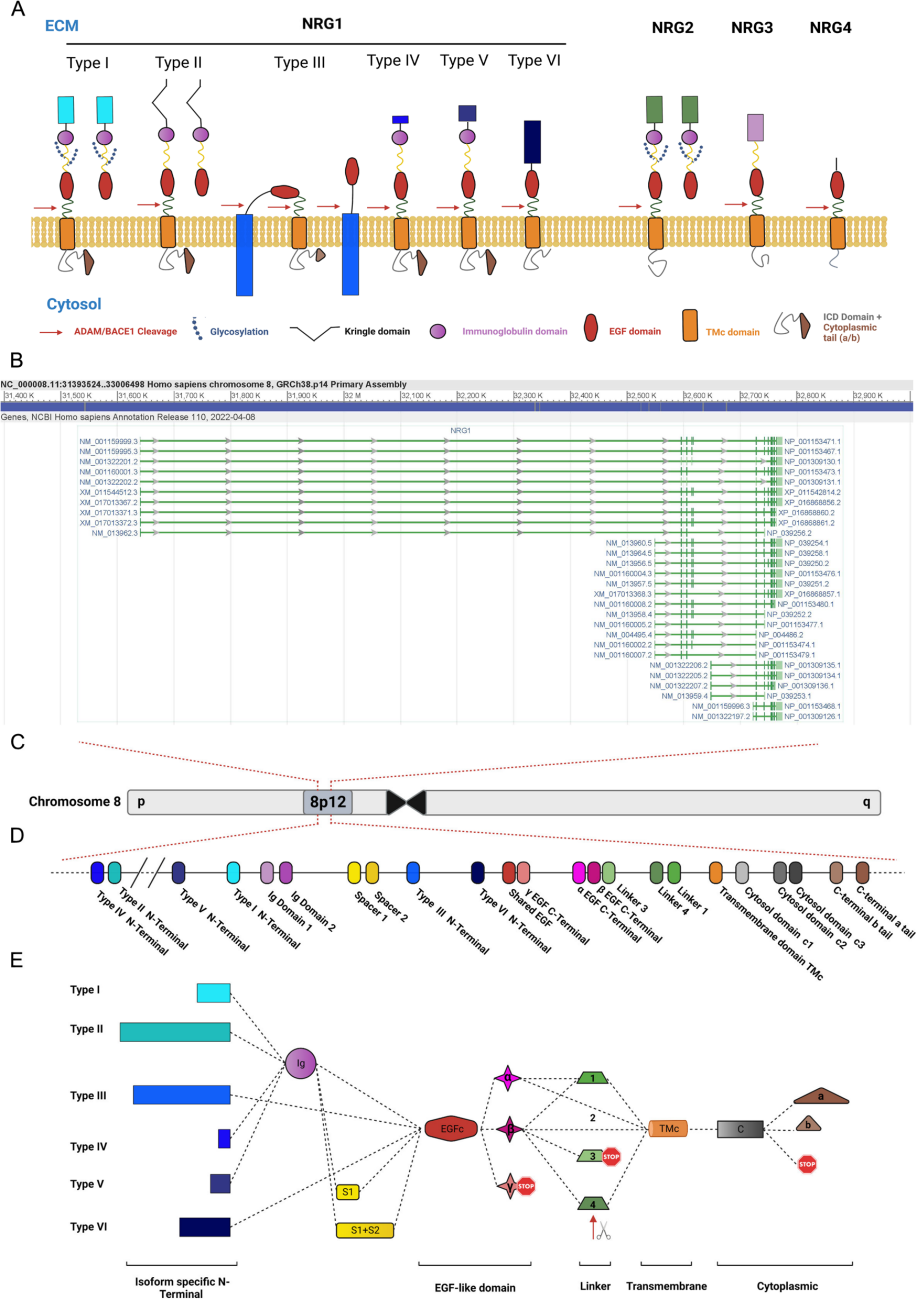
The human *NRG1* locus. **A** Representation of Neuregulin protein isoforms and their domain distribution. Note that in NRG1 classes IV, V and VI all isoforms that have been characterized are transmembrane, while I, II and III also present isoforms that don’t. Legend below the cell membrane describes symbols for ADAM/BACE1 cleavage site (red arrow), glycosylation site (blue chain), kringle domain (black V), IgG domain (purple circle), EGF domain (red oval), Transmembrane domain (orange rectangle), intracellular domain (grey squiggle, with or without brown triangle). N-terminal domains of NRG1 reference modularity shown in Fig. 1B and C, including the membrane-tethered N-terminal of NRG1-III isoforms. **B** Screenshot of the NCBI RefSeq curated products from the *NRG1* locus showing 28 protein coding transcriptional variants ([9] accessed 26/1/2023). Left and right labels correspond to transcript and protein accessions, respectively*.* **C** Representation of the NRG1 genomic locus (Chr 8p12). **D** Schematic annotating exons in the human *NRG1* locus with modular protein coding domains. **E** Schematic showing combinatorial protein domains for previously annotated NRG1 isoforms. Rows show the six major NRG1 classes, defined by alternate N-terminals. Columns show alternate domains: domains within a column are mutually exclusive. Dotted lines show known connections between domains in different NRG1 isoforms. Red stop symbols represent translation stop. Red arrows represent a protease cleavage point

Neuregulins (NRGs) are a family of highly pleiotropic growth factors derived from four paralogous genes (*NRG1-4*) (Fig. 1A). NRGs are typically synthesized as transmembrane pro-peptides that are cleaved by metalloproteases in the extracellular space to form a bioactive peptide with an exposed epidermal growth factor-like (EGF) domain that can bind erythroblastic leukemia viral oncogene homolog (ERBB) receptors. The human *Neuregulin-1* (*NRG1*) locus, on Chromosome 8p12, generates numerous isoforms (Fig. 1A-C) which are thought to be tissue-specific and functionally diverse [1]. NRG1 has been implicated in the development of multiple tissues by promoting cell division within the stem cell niche and in differentiation trajectories [2] including progenitor cells in the gut [3], skeletal muscle [4, 5] and cardiac cells [6], as well as nervous system development [7, 8]. These studies demonstrate NRG1’s key role in organogenesis and the importance of understanding how NRG1 isoforms exert tissue-specific effects to maintain the adult stem cell niche.

NRG1 and its receptors are involved in several diseases and targets for clinical research. Germline mutations in NRG1 are associated with developmental brain disorders such as schizophrenia [10] as well as degenerative disorders such as amyotrophic lateral sclerosis [11] and Alzheimer’s disease [12]. Constitutively activated isoforms of NRG1 are implicated in cancer, for which blocking the NRG1 isoform Heregulin or its receptors (ERBB) are effective clinical strategies against solid tumours [13, 14]. Deficiencies in NRG1 are associated with Hirshprung’s disease, leading to poor innervation of the gut [15, 16], as well as abnormal brain development and mental disorders like bipolar disorder or schizophrenia [17, 18]. In contrast, circulating NRG1 is associated with cardiac disease and morbidity following heart failure [19]. NRG1 is also involved in modulating the immune response [20, 21], controlling insulin related liver activity [22], cell migration [23] and cell–cell recognition and viability in the central nervous system [24]. Therefore, there are clear clinical benefits in understanding which cells express the different NRG1 isoforms in each tissue and how they play their tissue-specific roles in different biological processes.

The diversity of isoform functions in healthy development and disease across different organs is mirrored by the structural diversity and tissue specific transcriptional regulation of NRG1 products. The *NRG1* gene encodes at least 28 isoforms (Fig. 1B) according to the ENTREZ Gene reference transcript list [9, 25]. Others have reported higher isoform heterogeneity from this locus [26]. This extraordinary transcript diversity is due to the use of alternate transcription initiation sites, cassette exon usage, and alternate polyadenylation sequences. The locus is remarkably modular (Fig. 1D), which allows for different structural combinations of NRG1 domains depending on their start site (class I-VI), the use of alternative linker domains, which are the protease targets for ectodomain shedding (1–4), the type of EGF-like domain (α, β or γ), and whether the pro-peptide is membrane tethered or not (Fig. 1A, D). One well characterized isoform class, NRG1-III, contains a unique N-terminal domain that includes a sequence that locates to the cell membrane [27]. This means that upon NRG1 processing, the growth factor domain will not be released from the source cell but will function as a juxtracrine signal that allows neurons to establish key cell–cell interactions (with other neurons, oligodendrocytes, or muscle fibers) and regulates the survival of both interacting cells [24, 28]. In rare circumstances, another cleavage site between the N-terminal domain and the EGF domain can also be targeted by a protease, releasing the EGF domain to the ECM [29].

More recently, NRG1 has been described as an important factor regulating the stem cell niche in the gut [3]. In this context, NRG1 likely modulates stemness, proliferation and identity of progenitor cells in the niche, and it is required to recapitulate certain secretory and absorptive functions in human gut organoids [30]. Beyond development and tissue repair, NRG1 secreted by macrophages has also been implicated in inflammation [3, 31]. However, the specific NRG1 isoforms expressed by macrophages in these contexts have not yet been described.

Determining the sources and classes of different NRG1 isoforms is an important part of understanding how NRG1 directs these different developmental, reparative, and inflammatory outcomes. Here, data mining to assess the expression profiles of NRG1 led to the identification of a previously uncharacterized TSS that appears to be used exclusively in cells of the myeloid lineage. We propose that transcripts generated from this alternative TSS belong to a new NRG1 Class, NRG1-VII. Using Oxford Nanopore sequencing, we identified eight class VII isoforms with distinct transcript structures and predicted the protein characteristics of these isoforms. qRT-PCR targeting the unique first exon of NRG1-VII transcripts in human cells confirmed that class VII isoforms are expressed by monocytes, infiltrating macrophages and tissue resident macrophages. Immunohistochemistry using antibodies directed towards the EGF-like or intracellular domains (ICD) demonstrated that tissue-resident macrophages are a major source of NRG1 in these tissues, an observation further supported by transcriptional evidence derived from single cell data collated within the Human Protein Atlas. This study therefore contributes to untangling the complexity of this already intricate locus by characterizing the structure and distribution of myeloid-specific NRG1 isoforms.

## Results

### NRG1-VII is defined by a novel TSS discovered in myeloid cells

**Fig. 2:**
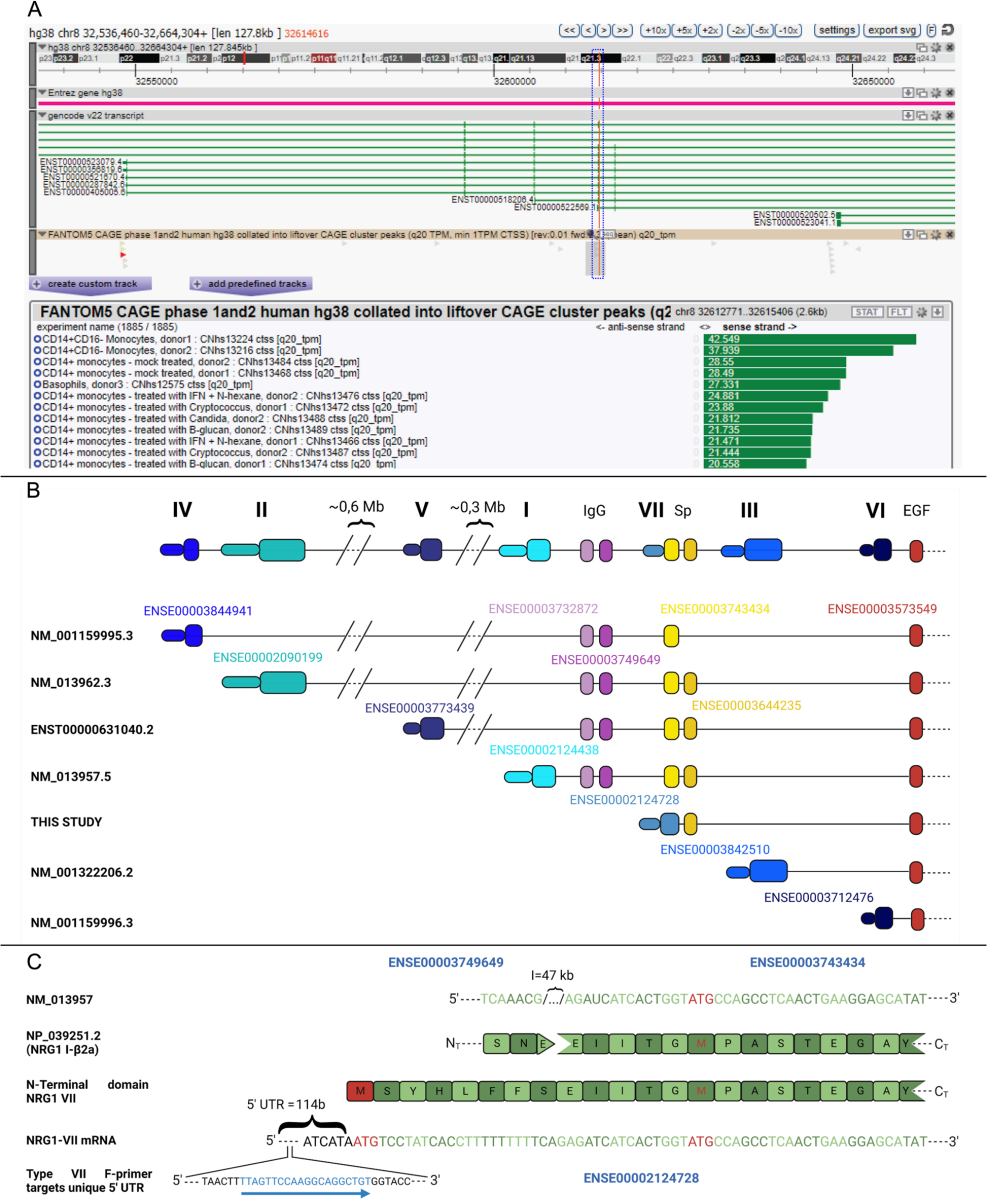
Identification of a myeloid specific TSS in the human NRG1 locus. **A** Screenshot of the ZENBU genome browser showing the Gencode v22 transcript track, (accessed 26/1/23 [33]) corresponding to the FANTOM5 phase 1 and 2 TSS peaks overlapping the starting exon of NRG1 class VII. Red vertical line encased in blue dashed box indicates the position for the TSS of interest. Tag selection (grey bar, FANTOM5 CAGE track) indicates region for tag quantification in the FANTOM5 experiment table. The table is truncated to show the top 6 samples are primary myeloid cells, representative of the top 57/115 samples with > 1TPM) Experimental samples are ordered by highest number of CAGE tags counted in this area on sense strand (green bars, showing counts as TPM). **B** Schematic of the human *NRG1* locus highlighting alternate transcriptional starting exons that correspond to seven isoform classes. The schematic shows 5’ exon composition until the first shared exon (containing the EGF domain). Exons are numbered by ENSEMBL accessions. **C** Alignment of mRNA and protein sequences of NRG1 class I and class VII to show an in-frame translation initiation of NRG1 class VII. Initiating methionine (M) or start codon (ATG) in red text. The blue arrow shows the target sequence of the forward primer for *NRG1-VII* mRNA’s unique 5’ UTR. The *NRG1-VII* transcript start is conserved in other mammalian genomes (Sup. Fig. 1)

Since the different isoforms of NRG1 have varying structures and binding affinities to their receptors, we sought to investigate which of these were expressed by myeloid cells. First, we used the Functional Annotation of the Mammalian Genome (FANTOM5) database [32], a catalogue that maps the TSSs of genes expressed in 975 human samples (including cells, tissues, and cell lines). Here, we found a previously uncharacterized TSS of NRG1 that was exclusively active in myeloid cells, including monocytes, macrophages, and basophils (Fig. 2A). A schematic of the locus suggested that this represents a potential new class of NRG1 transcripts which we prospectively named NRG1-VII (Fig. 2). The FANTOM TSS predicted that the NRG1-VII starting exon contained 139 base pairs (bp) of mRNA sequence unique to transcripts originating from this site, and an in-frame methionine was identified 115 bp downstream from the start of transcription (Fig. 2B, C). Therefore, we aimed to characterize whether this newly described TSS would lead to the expression of a new class of protein coding transcripts.

The TSS for NRG1-VII initiates in the intron positioned 5’ of ENSEMBL exon ENSE00003743434, which encodes for part of the spacer sequence between the Ig and EGF-like domains in the canonical NRG1 protein (Fig. 2B). The TSS adds a 5’ untranslated region (UTR) to the transcript that extends the exon from 51 to 190 bp (ENSE00002124728) and includes an initiating Methionine at nucleotide position 115–117 bp. This newly identified TSS generates a unique 5’ UTR for its transcripts. Within this 5’-UTR, a unique 20-nucleotide sequence was identified that allowed specific detection and amplification of the NRG1-VII isoforms and primers were designed to target it (Fig. 2C; Table 1). Isoforms arising from this new TSS would present a unique sequence of 8 amino acids (MSYHLFFS), a class VII specific N-terminal domain. This is followed by the 17 amino acids that are commonly present in this exon in other isoform classes (I, II, IV and V). This is the shortest known isoform specific N-terminal domain, and the only one that is present within an exon that other isoforms may include.

**Table 1:** Primers designed for qRT-PCR and Nanopore Long Read Amplicon Sequencing. Note that underlined regions of the primers correspond to the universal Oxford Nanopore Technologies (ONT) 5’mod sequence

Gene	F-Primer	Tm °C	R-primer	Tm °C
B2M	TAGCTGTGCTCGCGCTACT	66.5	TTCAATGTCGGATGGATGAA	60.2
NRG1 I	CAAAGAAGGCAGAGGCAAAG	62.9	AACTGGTTTCACACCGAAGG	63.9
NRG1 II	AACCTCAAGAAGGAGGTCAGC	63.9	AACTGGTTTCACACCGAAGG	63.9
NRG1 III	CCGACACCGAAGAATCGTAT	63.8	ACTCCCCTCCATTCACACAG	64.0
NRG1 IV	GCGACAGAGAGGGAGGA	63.6	AACTGGTTTCACACCGAAGG	63.9
NRG1 V	AATTCTTCTACGGAGTTTTAAGGTACAC	62.6	GCCGATTCCTGGCTTTTCAT	67.2
NRG1 VI	TCTTCAGGAACCACCTAAGCA	63.6	TCTCCTTCTCCGCACATTTT	63.6
NRG1 VII	TTAGTTCCAAGGCAGGCTGT	63.7	TTGCTCCTTCTGTGGATACTGA	63.7
CD11b	AGAACAACATGCCCAGAACC	63.9	GCGGTCCCATATGACAGTCT	63.9
CD14	GCCGCTGTGTAGGAAAGAAG	63.8	ATCGTCCAGCTCACAAGGTT	63.7
CD16	TGAGGTGTCACAGCTGGAAG	64.3	GGTTGACACTGCCAAACCTT	63.9
CD43	AGTGCTGCGTCCTTATCAGC	64.4	CAAACAGGCAGGAGCAAGAG	65.0
CD45	AGAATACTGGCCGTCAATGG	63.8	GCTGAAGGCATTCACTCTCC	63.9
SPL1	CCAGCTCAGATGAGGAGGAG	64.2	CAGGTCCAACAGGAACTGGT	64.0
NRG1-VII Nanopore	Forward amplicon: TTTCTGTTGGTGCTGATATTGC TTAGTTCCAAGGCAGGCTGT	84	Reverse short amplicon: ACTTGCCTGTCGCTCTATCTTCGATGCAGCAACAAGAAAGCA Reverse long amplicon: ACTTGCCTGTCGCTCTATCTTCTTTCCTGTTTTCTATTTGCAGAAC	79.4 83.9

We mapped the transcriptional activity arising from this TSS and identified 2 Expressed Sequence Tags (ESTs) that had been sequenced from the 5’ end (NCBI accessions BI908144 and BI907799) and originated from the *NRG1-VII* TSS. These originated from the same library (SAMN00164230) made from a pool of non-activated human leukocytes from anonymous donors. Both ESTs harbored evidence of an open reading frame (ORF) in frame with other NRG1 isoforms, but both were 3’ truncated. To further validate the potential activity of this TSS we looked for evidence this isoform class was present in other mammal species. Data available from 15 non-human primate species [34] and cross species alignment shows that the *NRG1-VII* TSS is present and highly specific to bone marrow and whole blood, while isoforms isolated from other tissues use alternate TSSs (Sup. Figure 1A). We also found transcriptional evidence of an equivalent TSS in *Mus musculus* and *Sus scrofa* derived from myeloid cells (Sup. Figure 1B,C) confirming that the class VII TSS and its expression in myeloid cells are conserved through evolution.

### NRG1-VII TSS transcribes at least 8 distinct high-confidence transcripts in myeloid cells

**Fig. 3:**
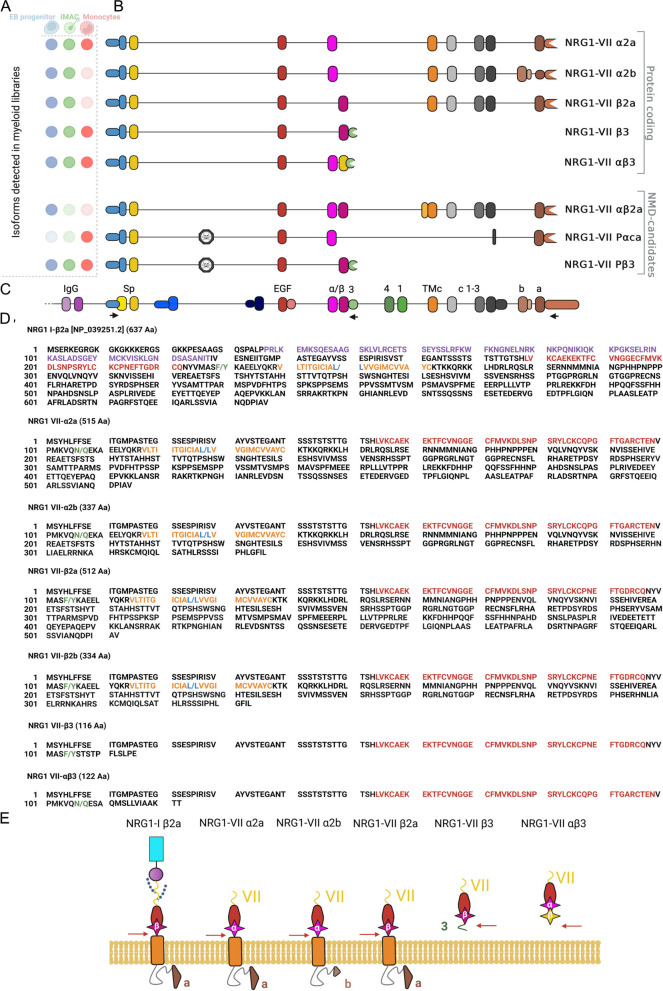
Sequence and structure of NRG1-VII isoforms (**A**) Representation of the different samples analyzed in the study and the presence or absence of the identified high-confidence transcripts in each of them (opaque = present, transparent = absent). Progenitors and iMACs are derived from PB001.1 ip (**B**) Representation of the exon structure for 8 NRG1-VII isoforms sequenced using Oxford Nanopore long-read sequencing and their designations based on exon combinations according to the locus nomenclature. **C** Human NRG1 locus reference with all known exons. Arrows show target regions for the single forward primer and both reverse primers used in the amplicon sequencing (see also Table 1). **D** Predicted translation of protein sequences of the newly characterized NRG1 class VII isoforms, compared to canonical NRG1-Iβ2a (top sequence). Text colour indicates known protein motifs: purple Ig; red EGF; orange transmembrane domains. “/” represents predicted pro-peptide cleavage points (green text represents ADAM/BACE1 proteolysis, blue represents γ-secretase proteolysis). Dashed lines in all panels represent sequences that are not shown. **E** Schematic of predicted translated proteins for NRG1-VII isoforms compared to canonical NRG1-I (Left), borrowing from domain schema shown in Fig. 1. Alternate (α or β) EGF domains annotated in pink. Intracellular triangles represent alternate a or b cytoplasmic tails. The yellow β domain represents the peptide sequence that arises from the related exon but is translated to a different peptide sequence due to a frame change

To characterize the diversity of *NRG1* transcripts that use the *NRG1-VII* TSS, we performed Oxford Nanopore long-read amplicon sequencing on in vitro and in vivo derived myeloid cells (Fig. 3A-E). We designed a forward primer that targets the 5’ UTR of *NRG1-VII* transcripts, which is unique to this class of NRG1 and does not overlap any other NRG1 isoform classes; additionally, two reverse primers were designed to target two of the known alternative transcriptional stops that we had previously validated as active in myeloid cells through PCR (Fig. 3C). We called amplicons “short” when the reverse primer targeted exon “β” and “long” for the reverse primer in exon “α”.

In total, we identified 8 novel high-confidence transcripts that were assigned at least 5% of the reads present in a sample from either the short or long amplicons (Fig. 3B, D), using the IsoLamp pipeline (see Materials and Methods). Each amplicon was studied independently in in vitro progenitors and differentiated macrophages, and in blood-isolated monocytes. Five of these transcripts had an open reading frame (ORF) following a start codon (ATG), in frame with all other previously described NRG1 isoforms, and no premature stop codons in early exons. We predict that these five transcripts could be protein coding (Fig. 3B), including two short and three long isoforms. The three long isoforms would include a transmembrane domain (NRG1-VII α2a, α2b and β2a) and likely undergo canonical processing through metalloproteases for the EGF-containing peptide to be released. On the other hand, the two short isoforms would lack the transmembrane and intracellular domains.

The short isoforms were detected in all myeloid libraries and lacked the transmembrane domain (Fig. 3E). Of these, NRG1-VII αβ3 contained both α and β EGF-like exons, a combination that had been previously captured in refseq NM_004495, which we now show was a truncated transcript as its TSS had not been defined. This resolves the full-length sequence of the Class VII αβ isoforms. The presence of both α and β domains in NRG1-VII αβ3 introduced a frame shift in the β exon, changing the peptide sequence of this isoform to a unique C-terminal end. This feature might affect the binding dynamics of these isoforms to ERBB receptors, making it of special interest.

Monocytes expressed both short isoforms, but only expressed one long isoform, NRG-VII α 2a. Two additional isoforms were detected in the monocyte library that we predict are sensitive to nonsense mediated decay (NMD). Transcripts NRG1-VII Pαca and NRG1-VII Pβ3 include a previously uncharacterized exon (Fig. 3A) located in intron 2 of the other class VII transcripts. Additional support for this exon can be found in isoforms present in smaller proportions like P-α2a (Sup. Figure 2) This exon spans 111 bases (chr8:32,676,084–32,676,195). We identified this as a poison exon, as it introduces an amber stop codon (TAG), resulting in the following amino acid sequence:

- MSYHLFFSEIITGMPASTEGAYVSSESPIRISVSTEGANTSSFITDECCHGGQYHNTAKSICLILMF-.

A third NMD candidate was identified in the iPSC-derived myeloid progenitors, NRG1-VII αβ2a. For isoform αβ2a, this combination introduces an early stop codon that we theorize would lead the transcript to nonsense mediated decay. All three NMD-candidate transcripts passed our high-confidence transcript filters. We manually removed additional isoforms that passed our analysis threshold but had less than 5% read coverage. These isoforms varied only in a few bases across a splice junction (Sup. Figures 2– 3) and were detected in only one library and were therefore considered as likely sequencing artefacts. There are two possible exceptions: NRG1-VII β2b and P-α2a. Isoform β2b was detected in both iPSC derived progenitors and iMACs, and P-α2a was found in monocytes, at levels between 1 and 5% (Sup. Figure 2).

### Macrophages are a major source of NRG1 in human tissue

**Fig. 4:**
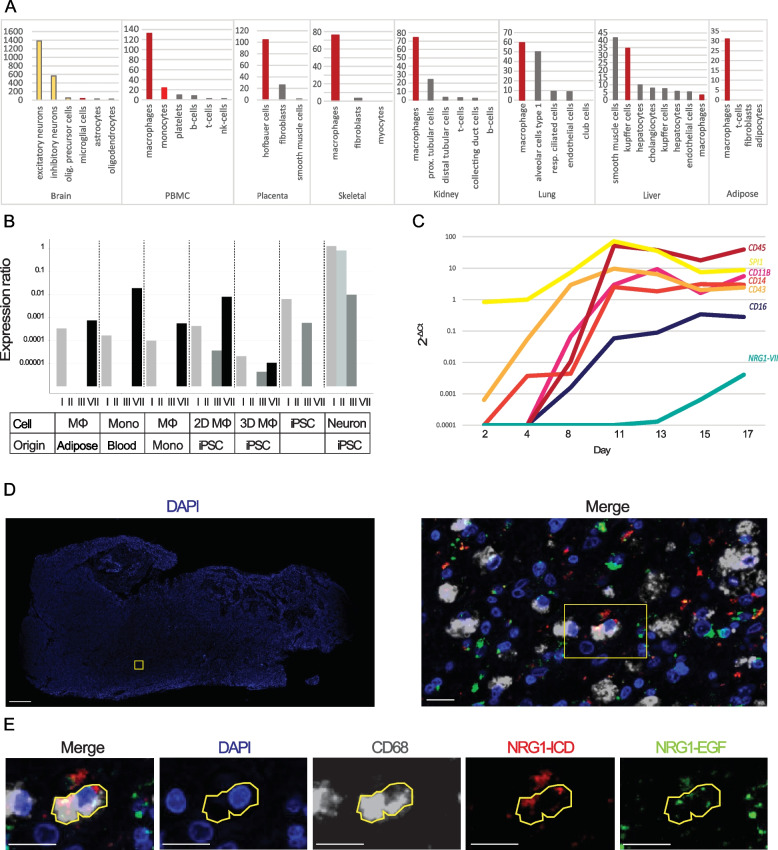
NRG1 expression profile in myeloid cells. **A** Quantification of NRG1 mRNA expression in different human tissues according to The Human Protein Atlas [35]. A plethora of representative tissues that express NRG1 are shown here. The y axis represents nTPM (Number of transcripts per million). Neurons shown in yellow, myeloid cells in red, other cell types in grey. **B** qRT-PCR data measuring presence of the different NRG1 classes in different cell types. Classes IV-VI were not detected in any of the samples. Expression ratios include Pfaffl primer efficiency [36]. Mono = Monocyte, Mφ: macrophage, iPSC: Induced pluripotent stem cell. Monocytes, monocyte derived macrophages and adipose tissue macrophages were isolated from patients. 2D macrophages and cortical neurons are derived using Kolf2.1 iPSCs. iPSCs and 3D macrophages are PB001.1 cells. **C** Time series showing qRT-PCR expression of typical myeloid markers in progenitor maturation (PB001.1) and relationship with myeloid specific NRG1-VII transcripts. **D** Immunostaining of GBM showing presence of CD68^+^ cells that express NRG1 peptides in vivo. The left panel shows sample section stained with DAPI. The boxes indicate the regions shown in the right panel in D and the cell in E, respectively. Scale bar on left panel is 1 mm, scale bar on right panel represents 20 μm. **E** Example of cell in which all markers (CD68, NRG1-EGF and NRG1-ICD) are expressed. Scale bars = 20 μm

We next assessed the distribution patterns of NRG1 in single-cell RNA-seq experiments in the Human Protein Atlas [35], which revealed that the *NRG1* locus was actively expressed in a large variety of human tissues (Sup. Figure 4). Single cell expression data revealed NRG1 activity in different cell types, such as neurons in the brain and eye; glandular stromal cells in colon, ovary, or endometrium; endothelial cells in the heart and liver; and epithelial cells in the kidney and lung (Fig. 4A). In most of these organs, macrophages are the main source of NRG1 (Fig. 4A, Sup. Figure 4). One outstanding exception is the brain, where neurons show the highest levels of NRG1 expression in the human body. However, no isoform-specific single cell data is currently available to compare differential isoform expression between the different macrophage types present across the tissues investigated.

### NRG1-VII expression in myeloid cells is affected by differentiation and maturation

We sought to confirm if isoform VII is the only or even primary TSS used by myeloid cells. Therefore, using primers that could discriminate between each unique start exon (Table 1), we investigated the patterns of the seven classes of NRG1 isoforms in different in vivo and in vitro myeloid cells (Fig. 4B). Expression of NRG1-VII was detected in all myeloid cells, and it was the isoform class showing highest expression for cells that belong to this lineage (except for macrophages derived from a 2D epithelium). In contrast, control cell types (hiPSCs and iPSC-derived cortical neurons) showed high expression of other *NRG1* classes, but not NRG1-VII.

Having demonstrated that our iPSC derived myeloid cells can be used to model the use of the class VII TSS, we next investigated at which point in the differentiation of iPSC to macrophages the expression of NRG1 VII emerged. Figure 4C shows the qPCR results tracking early commitment to blood using the myeloid transcription factor SP1 and hematopoietic E-selectin CD43, which are detectable within 2 days of growth factor culture. The blood-associated tyrosine phosphatase CD45 was detected at the same time that myeloid maturation markers integrin CD11B and pathogen receptor CD14 were also identified. CD16 marks expression of the FcR family which are typically seen in mature, phagocytic cells and this is one of the latter markers to emerge in our iPSC-macrophage differentiation. NRG1-VII is detectable several days after the emergence of these mature macrophage markers and continues to increase in the cultured macrophages. This is consistent with the observation that alternate differentiation of iPSC (2D-derived) macrophages, which were matured for up to three weeks in culture expressed higher levels of NRG1-VII compared to the embryoid body derived macrophages (3D, Fig. 4B) which were sampled one week after macrophage derivation. Although iPSC-macrophages do not transition through a monocyte stage, monocytes are precursors to tissue macrophages, so it was interesting to observe higher levels of NRG1-VII in primary monocytes than monocyte-derived macrophages.

### NRG1-VII expression in myeloid cells is affected by differentiation and maturation

To confirm that NRG1 mRNAs are translated into proteins in myeloid cells, we then performed immunohistochemical staining of NRG1 in human glioblastoma (GBM) tissue which is enriched in bone marrow-derived macrophages [37]. Myeloid cells were identified using a CD68 antibody; antibodies detecting the extracellular (EGF-like domain) and intracellular (ICD) domains of NRG1 were used. NRG1 was detected in both myeloid and non-myeloid cells (Fig. 4D,E). Not all CD68 + cells expressed NRG1, and those cells that did exhibited different patterns of expression (Sup. Figure 2). Altogether, these results show that myeloid cells express NRG1 peptides in human tissues but are not the sole contributors to the NRG1 pool in the brain. They also reveal diverse NRG1 expression patterns in myeloid cells, which may be consistent with the different isoforms observed in our sequencing libraries, or consistent with active processing of membrane bound-NRG1.

## Discussion

Macrophages have been reported as a major source of NRG1 in different human tissues [35], including a recent report highlighting the importance of NRG1 in the developing gut [3]. Our investigation of *NRG1* expression in the public single cell atlases, including the Human Protein Atlas mRNA dataset, suggested that macrophages are a major source of NRG1 in most tissues. However, the specific isoforms involved in the macrophage mediated NRG1 secretion were uncharacterized. Here we report that myeloid cells preferentially use a novel TSS that is myeloid-specific and conserved which generates a previously uncharacterized class of NRG1 isoforms. The tissue variability and functional versatility of NRG1 are a consequence of the alternative promoter usage and exon retention that give rise to a high diversity of isoforms.

Transcripts arising from the human *NRG1* locus are differentially regulated by tissue type or developmental stage. Control over the six known isoform classes is achieved through differential proximal promoter usage at six unique TSSs, each of which is controlled by different transcription factors [38]. All available data indicates that the use of this TSS is exclusive to the myeloid lineage. The concrete mechanisms and transcription machinery involved in the process are yet to be described. Additional transcript processing can lead to alternative exon usage and define the functional modules in the protein, like the linker or the cytoplasmic tails (Fig. 1 D, E), mechanisms that are tissue-specific [39]. The diversity of NRG1 isoform classes and their specificity of expression in both a temporal anatomical and cell-specific manner, suggest distinct roles of different isoforms in tissue patterning, especially in different brain regions [40]. Further characterization of each of the NRG1-VII isoforms is hence needed to elucidate their regulation and functional diversity.

It was previously reported that the highest NRG1 expression levels in human tissues was in blood plasma [41, 42]. This is unsurprising, as maturation of myeloid cells in vivo seems to cause the downregulation of this gene across the monocyte-macrophage differentiation axis (Fig. 2A), with a subset of macrophages expressing NRG1 in tissues (Fig. 4). Our review of the FANTOM and The Human Protein Atlas suggests that monocytes are the main source of NRG1 in blood. The function of monocyte-derived NRG1 has not yet been described, however, circulating levels of NRG1 correlate with liver metabolic activity [22] as well as post-infarct recovery and cardiovascular health improvement [43]. This suggests that circulating NRG1 could play an important role in hepatic and cardiac health.

We further found that monocytes express at least five high-confidence NRG1-VII isoforms, including transcripts that contain a novel ‘poison’ exon (NRG1-VII Pαca and Pβ3) predicted to prematurely terminate translation (Fig. 3A). We also note the unusual exon composition of isoform NRG1-VII Pαca, indicating it could be a PCR artifact; thus, further validation might be required on this isoform, despite its amplicon constituting over 15% of the detected expression. Additional isoforms containing the monocyte poison exon like NRG1-VII Pα2a were also found, but in much lower proportions (Sup. Figure 2). However, this supports the use of the poison exon by monocytes. This exon introduces an early stop codon and hence is likely to drive the transcript to nonsense mediated decay, generating a monocyte specific transcriptional mechanism to regulate the levels of NRG1 synthesis. Due to the high levels of expression of this gene in monocytes compared to other cell types, we hypothesize that these represent regulatory mechanisms that allow control over the levels of NRG1 expression in circulation.

Neuregulins interact with monomeric ERBB proteins (ERBB1-4), via an EGF-like domain, promoting dimerization and trans-phosphorylation of these receptors. Each ERBB monomer can bind different EGF members with differential ligand binding affinities, but only ERBB 3 and 4 can bind Neuregulins. Though NRG1 and NRG2 can bind to both receptors as monomers [44], NRG3 and NRG4 only bind ERBB4 monomers [45, 46]. Dimerized receptors can discriminate between different isoforms of NRG1, which is evidenced by differential phosphorylation on receptor tyrosine residues [47, 48]. Dimerization leads to the recruitment of different adaptor proteins, notably GRB or PI3K, which then trigger specific secondary cascades and regulate cell activity [49]. NRG1-VII isoforms present a very short N-terminal domain compared to most other isoforms. It has been previously reported that the size and structure of the NRG1 N-terminal domain can dictate the receptor availability and recycling, affecting internal phosphorylation and internal signaling in the receiving cell [50]. Additionally, the discovery of isoform NRG1-VIIαβ3 as a coding isoform, adds a novel sequence possibility in the EGF domain. This is likely to determine the protein’s binding dynamics and internal phosphorylation, hence changing its functional properties. Functional validation is necessary to confirm whether this isoform type has unique properties given its EGF domain structure.

NRG1 is involved in many different functions, attributed to the many isoforms which are tissue-restricted during different developmental stages. Further, while NRG1 is conserved at the protein level, the function of specific isoforms has been shown to differ between species. For example, heart trabeculation failure, which leads to a lethal phenotype in mice at day 10.5, is a phenotype common to NRG1-/-, ERBB2-/- and ERBB4-/- mice [6, 51]. Although 11 different isoforms have been identified in the heart at this developmental stage, the phenotype can be attributed specifically to the absence of β-EGF types I and II, which are the isoforms containing the immunoglobulin (Ig) domain [52]. However, the opposite is observed in zebrafish where Ig-like neuregulin-1 isoforms are dispensable for this process [53]. Thus, while essential processes in development can be attributed to specific subtypes of NRG1, differences between species showcase the need to characterize the different human isoforms using appropriate cell type and developmental models.

NRG1 and the ERBB receptors are important clinical target in different cancer types. For example, NRG1 fusion proteins cause pathological activation of ERBB receptors [54]. NRG1 expression correlates with a shorter survival in patients with glioma [55], which could be related to its role in cell migration and proliferation. Glioblastomas particularly exhibit high macrophage infiltration [56]. Our results show that tumor-associated myeloid cells (CD68^+^) may contribute to the NRG1 pool seen in these tumors. Due to the lack of unique domains in class VII isoforms, the antibodies used in this study targeted common NRG1 regions (the EGF-like and intracellular domains). Even though the available antibodies lack specificity to prove that class VII isoforms are translated in this disease, the only isoforms detected in primary monocytes or macrophages in this study belong to classes I and VII.

While NRG1 double positive myeloid cells may express newly synthesized pro-peptides, we also observed CD68^+^/NRG1-ICD^+^ cells indicating that the EGF-like domain had been cleaved. Alternatively, the presence of CD68^+^/NRG1-EGF^+^ cells suggests that some cells restrict their expression only to isoforms that end transcription in linker 3 and lack a transmembrane domain. Therefore, we confirmed that macrophages in vivo express NRG1 and that there are distinct populations of macrophages in tissue based on their NRG1 expression patterns.

To determine whether in vitro derived myeloid cells can be used to model the activity of the NRG1-VII TSS, we used primers designed to target specific TSS usage in different cell types. We confirmed that NRG1-VII was expressed in all myeloid samples and in no others, suggesting the TSS is active. However, iPSC-derived samples showed expression of NRG1-III. This could be a result of incomplete differentiation of the culture, or retention of stem cell features in in vitro derived myeloid cells. Moreover, the time series data obtained during the differentiation process shows that NRG1 expression follows mature markers like CD16; in vivo, NRG1 expression precedes CD16, and as CD16 is upregulated, NRG1 expression decreases. Hence, while we show activity of the TSS, the in vitro model may not recapitulate the transcriptional and maturation sequence seen in vivo due to the differences in the differentiation process.

Description of a new TSS class, NRG1-VII, including at least five new protein-coding isoforms, has expanded the known NRG1 protein coding isoforms from 28 to 33. This study adds eight new transcripts that are specific to myeloid cells. However, it is likely that the full transcriptional profile of this locus has not yet been described; for example, while long-read amplicon sequencing is a sensitive isoform recovery method [57], it is limited by the primer set(s) used. Thus, only NRG1-VII isoforms utilizing linker 3 or the “a tail” regions were amplifiable in this study. It is highly likely that NRG1-VII isoforms using the 3’ end present in exon c3 (ENSE00002109887) also exist, which should be validated with additional work.

Here we showed that myeloid cells exhibit a unique regulation pattern of the NRG1 locus to generate cell specific isoforms, potentially playing an important role in diverse diseases. Only through a thorough investigation of this locus can we better understand each process and develop clinical strategies to prevent or treat the different pathologies associated with this locus. Further detailed investigation on the molecular genetic features and functions of these novel isoforms might uncover how NRG1-VII isoforms elicit differential receptor activity and downstream effects as previously described for other NRG1 isoforms. This could lead to targeted therapies and an improved understanding of the complexity of the in vivo system, helping us recreate the processes in which appropriate signals are essential to model the desired biological mechanisms.

## Materials and methods

### Cell lines

The use of existing stem cell lines was performed in accordance with The University of Melbourne ethics committee HREC (approval 1851831).

The line of human induced pluripotent stem cells (iPSC) used were: PB001.1 [58], obtained from the Stem Cell Core Facility at the Murdoch Children’s Research Institute; and Kolf2.1 (hPSCReg accession WTSIi018-B; Welcome Trust Sanger Institute). Cortical neurons were differentiated from Kolf2.1 cells under University of Melbourne/Florey Institute for Medical Research Ethics ID: 12374. iPSC-derived macrophages were conducted using Kolf2.1 and PB001.1 cell lines in accordance with The University of Melbourne ethics committee HREC (approval 1851831). Monocytes were isolated from buffy coat, which was obtained from the Australian Red Cross Blood Service in accordance with The University of Melbourne ethics committee HREC (approval 1646608). Ethics for adipose tissue derived samples was obtained from the University of Melbourne Human Ethics Committee (ethics ID 1851533) and approved by The Avenue Hospital Human Research Ethics Committee (Ramsay Health; ethics ID WD00006, HREC reference number 2019/ETH/0050). For human glioblastoma samples, human ethics approval was covered by project application 1853511, approved by the Medicine and Dentistry Human Ethics Sub-Committee, The University of Melbourne.

### Stem cell culture

PB001.1 iPSC were cultured in Gibco™ Essential 8™ media with Essential 8™ supplement (Thermo Fisher Scientific; A1517001) on growth-factor reduced Matrigel® Matrix (Corning®; 356234) coated dishes. Kolf2.1 iPSCs were cultured in mTesR™1 basal medium (Stem cell Technogies™; 85850) on rhLaminin-521 (Thermo Fisher; A29249) coated plates. Cells were cultured with daily changed fresh media. Cell culture was performed in an APT.line™ C150 (E2) CO_2_ manual incubator (BINDER; 7001–0172) in constant and stable conditions of humidity, temperature (37 °C) and CO_2_ concentration in air (5%).

Cell passaging was performed routinely when cell confluency reached (70–80%). Cells were first washed with Gibco™ PBS (Thermo Fisher Scientific; 10010023). Then, a dilution of sterile 0.5 M EDTA (Thermo Fisher Scientific; 15575020) in said PBS at a concentration of 0.5 mM was used to detach the cells from the plate. Alternatively, ReLeSR™ (Stem Cell Technologies; 100–0483) was used for iPSC passaging according to the manufacturer’s instructions. After 3–4 min in humid incubator conditions, cells were collected and replated with fresh media.

### iPSC derived macrophages

iPSC-derived macrophages were differentiated as previously described [59]. Human iPSCs were differentiated into macrophages [60], but with the following alterations to the protocol: harvested cells were cultured in MAGIC media, which was changed as indicated in (Ng et al., 2008). For the 3D (Embryoid body) differentiation process, PB001.1 iPSC were plated in 10 cm non-treated Petri dishes (IWAKI; 1020–100) and placed on an orbital shaker (N-Biotek orbital shaker NB-T101SRC) in a humidified incubator with 5% CO_2_ at 37 °C. For the 2D differentiation process, Kolf2 iPSC were plated in a 10% solution of methyl cellulose in E6 media (Thermo Fisher, Cat. No. A1516401) with the same growth factor components as above. The 3D approach yielded progenitors within 7–11 days of culture, whereas progenitors were collected between day 9–20 for the 2D approach.

After at least 11 days of culture, cells can be observed detaching from the embryoid bodies or endothelial sheet, remaining in suspension in the media as non-adherent cells (which are characterized as myeloid progenitors). When all cells are collected and allowed to settle in a 15 mL Falcon tube (Corning®; CLS431470-500EA), embryoid bodies pelleted in the bottom, but progenitors stayed in suspension in the supernatant, and could then be collected. The supernatant was then centrifuged (Heraeus Multifuge 1S-R) at 400 rpm for 5 min to separate the progenitors from the media. Progenitors were then resuspended in macrophage maturation media, consisting of a 10% dilution in volume of FBS and 100 ng/mL CSF-1 (R&D Systems; 216-MC-500) in Gibco™ RPMI-1640 media (Thermo Fisher Scientific; 11875093). Cells were plated in Costar® 6-well tissue-culture treated plates (Corning®; 3516) for 4–7 days in stable incubator conditions (humid, 5% CO_2_, 37 °C), when cells showed morphological and molecular features displayed by macrophages.

Samples were collected for sequencing at the iPSC, progenitor and mature macrophage stages. iPSC and macrophages were collected from plates using the cell passaging approaches described above. Progenitors were collected directly from suspension cultures, pelleted via centrifugation at 400 g for 5 min at 4 °C. The cell pellet was used immediately for RNA isolation.

### iPSC derived cortical neurons

Kolf2.1 hiPSCs were cultured under xenogenic conditions as defined in [61]. The cells were then differentiated into cortical neurons following previously described protocols [62, 63]. Briefly, Cells were fed cortical base medium supplemented with 100 nM LDN193189 (Tocris; 6053) and 10uM SB431542 (Tocris; 1614) daily, from day 1 to 10 (inclusive). On day 11, cells were plated at a 4:10 split ratio in Cortical (CTX) base medium with Rocki, SB431542 and LDN193189, onto rhlaminin-521-coated 48 well plates. Cells were fed on day 12 and every alternate day until 18 using CTX base medium supplemented with rhFGF (R&D systems; 233-FB-010) at 20 ng/ml. Differentiating cultures were passaged in these conditions as necessary, at a 1:2 split until day 32 when 1.5 × 10^6^ cells were seeded onto poly-L-orthine/Laminin coated plates. Cells were collected for RNA extraction at day 42.

### Human samples and cell sorting

#### Blood monocyte isolation

Buffy Coat was obtained from the Australian Red Cross Blood Service. The blood was diluted with PBS at a 1:3 dilution and underlaid with Histopaque®-1077 (Sigma-Aldrich; Cat. No. 10771-100 ml). The underlaid blood samples were centrifuged (TECHCOMP CT1SRT) at 350 g for 30 min at 24 °C with no brake. Peripheral blood mononuclear cells (PBMCs) were isolated from the interphase and washed twice using MACs buffer (Gibco™ Dulbecco's phosphate-buffered saline (DPBS) (Ca2 + Mg2 + free) (Thermo Fisher Scientific; Cat. No. 14190144) with 0.5% heat inactivated Fetal Bovine Serum (FBS) (Thermo Fisher Scientific; Cat. No. 10082147 or 10099141) and 2 mM EDTA (Invitrogen™ UltraPure™ 0.5 M EDTA (Thermo Fisher Scientific; Cat. No. 15575020)) and centrifuging at 400 g for 5 min at 4 °C. Cell count and viability were determined using 0.4% Gibco™ Trypan Blue (Thermo Fisher Scientific; Cat. No. 15250061) using a hemocytometer. Cells were centrifuged at 400 g for 5 min at 4 °C and resuspended in 40 μl MACs buffer per 10^7^ cells. Monocytes were positively selected by a magnetic field using Human CD14 MicroBeads (Miltenyi Biotec; Cat. No. 130–050-201) and LS Columns (Miltenyi Biotec; Cat. No. 130–042-401), then cryopreserved in 10%DMSO, 20% fetal bovine serum, 70% RPMI until required. Prior to RNA isolation, cells from three individual donors were thawed in RPMI + 10% FBS for 4 h, then pooled for centrifugation at 400 g for 5 min at 4 °C. The cell pellet was used immediately for RNA isolation and RNA sequencing.

##### Adipose tissue samples

Adipose tissue (AT) was obtained and processed within biosafety cabinets as previously described (Raajendiran et al., 2019). The AT was rinsed with phosphate-buffered saline (PBS), blot-dried and transferred to a falcon tube containing RPMI media (ThermoFisher Scientific Australia) supplemented with 3% (w:v) bovine serum albumin (BSA) (Sigma Aldrich, Australia), 1 mg/ml Type 2 Collagenase (Sigma Aldrich, Australia) at a ratio of 0.5 g /2 ml to digest the extracellular matrix. The adipose tissue was minced into ~ 10 mg pieces using autoclaved scissors, and the falcon tube was transferred to a 37 °C shaking water bath for 45 min. The undigested AT was removed by straining the solution through 200 µm nylon filters (Corning, Australia), and the flow through containing the liberated cells was centrifuged for 10 min at room temperature (RT). The floating adipocytes were removed, and the pelleted stromal vascular fraction (SVF) was resuspended in 0.02% EDTA-PBS (Sigma Aldrich, Australia) to restrict cell clumping and transferred to an Eppendorf tube. The SVF was washed with 1 mL of 0.02% EDTA-PBS (Sigma Aldrich, Australia) and centrifuged at 2500 rpm for 2 min at RT. Following this, the supernatant was discarded, and the cells were gently resuspended in 1 mL of RBC lysis buffer (Life Technologies Cat# A1049201) for 15 min to lyse the RBCs. The cells were then pelleted following centrifugation at 2500 rpm for 2 min. The supernatant was removed, and the pellet was washed with PBS-EDTA and centrifuged at 2500 rpm for 2 min at RT. This process was repeated 3 times. The sample was then placed on ice, washed with ice-cold PBS-EDTA supplemented with 2% BSA (FACS buffer). The cells were then resuspended in 2 mL of FACS buffer, and the aggregated cells were removed through filtration through 40 µm filters (Corning, Australia). The cell flow-through was blocked for 2 h with FACS buffer on ice. The cell viability was calculated using Trypan Blue solution (ThermoFisher Scientific Australia) to determine the antibody concentration required for staining (CD45 5 µl / 1,000,000 live cells). Unstained and single fluorophore staining was performed on 250,000 cell aliquots to account for fluorescence spillover) was performed for 15 min on ice in the dark. The remaining SVF cells were incubated with CD45 and CD11b antibodies (1:100) for 15 min on ice in the dark. Five minutes prior to cell acquisition, propidium iodide (Sigma Aldrich, Australia) was added to account for cell viability.

### RNA extractions and cDNA synthesis

Total RNA was extracted using the RNeasyⓇ Plus Mini Kit (Qiagen; 74134) according to manufacturer’s instructions. After final total RNA elution in RNase-free water, overall RNA quality and concentration were measured using an RNA ScreenTape (Agilent Technologies; 5067–5576) in an Agilent 2200 Tapestation System (Agilent Technologies; G2964-90003).

cDNA synthesis was then performed using the isolated total RNA and considering the concentration values assigned to each of the samples for the coming steps. cDNA was synthesized using SensiFASTTM cDNA Synthesis Kit (BioLine; BIO-65053) and following all protocol specifications from the vendor. cDNA concentration and quality were checked using a D5000 ScreenTape (Agilent Technologies; 5067–5588) in an Agilent 2200 Tapestation System. Final cDNA samples were then stored at -20 °C.

### q-RT PCR

For mRNA quantification, the Applied Biosystems Viia7 ™ real time system was used (Thermo Fisher Scientific; 4453536) using a Fast 96 well plate hardware set up. The reactions in each well of the Micro Amp Fast 96 well reaction plate 0.1 mL (Thermo Fisher Scientific; 4346907) were prepared as indicated for the Fast SYBR™ Green Master Mix (Thermo Fisher Scientific; 4385612). Primers used in the reaction were designed for each transcript class of interest (Table 1). Plates were then sealed using Optical adhesive covers (Thermo Fisher Scientific; 436 0954). For quantification, *n* = 3 technical replicates were used.

Efficiencies (E) for each primer pair were calculated from serial dilutions of template and ranged between 95 and 98% for all NRG1 isoforms classes reported in results. Isoforms IV, V, VI were not detected in any samples. B2M was used as House Keeping gene (HKG) for all samples.

Quantification was determined using the Pfaffle method [36], where E is efficiency, HKG refers to the housekeeping gene, and GOI refers to the gene of interest (NRG1 isoform class of interest). CT is the cycle threshold of the logarithmic phase of amplification. Delta CT (Formula 2) was substituted for CT where comparisons between samples were made for the same primer pair.

1 $$\text {Relative}\;\text {expression}=\frac{{E\left(GOI\right)}^{CT\left(sample\right)}}{{E\left(HFG\right)}^{CT\left(sample\right)}}$$

2 $$\text {Relative}\ \text {expression}= \frac{{E\left(GOI\right)}^{CT\left(sample1-sample2\right)}}{{E\left(HFG\right)}^{CT\left(sample1 - sample2\right)}}$$

### Nanopore amplicon sequencing

NRG1-VII was amplified using the LongAmp® Taq 2X Master Mix (New England Biolabs; Cat #: M0287S) and the specified primers (Table 1) for either 30 cycled for the shorter amplicon or 40 cycles for the longer amplicon. The samples used were a pool (cells from independent biological replicates) of monocytes from 3 different blood donors, a pool of myeloid progenitors derived in vitro and macrophages differentiated from said in vitro derived progenitors. Target sequences were then amplified using specific primers (Table 1) and purified using AMPure beads (Beckman Coulter; A63880) at concentrations appropriate to each of the target sizes. Then, 2 ng of purified cDNA from each sample were barcoded following the EXP-PBC096 protocol from Oxford Nanopore Technologies. Samples were pooled (equimolar) and a sequencing library was prepared as described in the SQK-LSK110 Oxford Nanopore protocol. Samples were then loaded on a Flongle flow cell (FLO-FLG001) and sequenced using a GridION device.

### Sequencing data analysis

Basecalling was performed using Guppy (v6.3.8) using the super-accurate basecalling config file with a Qscore threshold of 10. To identify NRG1 Isoforms we used IsoLamp v1.0 [64] (https://github.com/ClarkLaboratory/IsoLamp), a bash pipeline for the identification of known and novel isoforms from targeted amplicon long-read sequencing data generated with Oxford Nanopore technologies. Briefly, IsoLamp takes passed Nanopore reads and maps them to a reference genome using Minimap2 [65]. Alignments that are highly accurate (> 95%), are full-length and have high accuracy splice junctions (> 90%) are used for isoform discovery with Bambu (v3.2.4) [66]. Isoform quantification is then performed with Salmon v0.14.2 [67]. IsoLamp was run in de novo mode setting BambuAnnotations = NULL. Each sample was run independently through the IsoLamp pipeline with ‘downsample reads’ parameter set to FALSE. All other parameters were set to default. Isoform annotation files and count matrixes were visualized using IsoVis (https://isomix.org/isovis) [68]. 

### Automated multiplex immunohistochemistry

Formalin-fixed paraffin-embedded (FFPE) GBM tissue sections were stained using the Bond RX automated stainer (Leica Biosystems). Slides were deparaffinized in xylene followed by exposure to a graded series of ethanol solutions for rehydration. Heat-induced epitope retrieval was performed with either a Citrate pH 6 buffer or Tris Ethylenediaminietetraacetic acid (EDTA) pH 9 buffer. Slides were blocked with 3% hydrogen peroxide (H_2_O_2_) to block endogenous peroxidase activity. For multiplexed IHC staining the Opal 6-plex Detection Kit (Akoya Biosciences) was used. Serial multiplexing was performed by repeating the sequence of antigen retrieval, primary antibody, and Opal polymer incubation, followed by Opal fluorophore visualisation for all antibodies as follows. GBM tissue was stained with CD68 (Abcam ab955, 1:100), NRG1 ICD (Abcam ab191139, 1:200) and NRG1 EGF-like domain (ThermoFisher; MA4-12896, 1:100). Slides were incubated for 1 h at room temperature with primary antibodies diluted in 1 × Opal blocking/antibody diluent (Akoya biosciences). Slides were subsequently incubated with the Opal Polymer HRP Ms + Rb secondary polymer for 30 min prior to incubation with Opal fluorophores (Opal 520, 540, 570, 620, 650 and 690) diluted at 1:150 in 1 × Plus Automation Amplification Diluent (Akoya biosciences) for 10 min. Slides were counterstained with 10 × Spectral DAPI and coverslipped with ProLong Glass Antifade Mountant (Invitrogen). Multispectral images were acquired at 20 × and 40 × magnification using PhenoImager ™ HT (Akoya Biosciences). inForm 2.4.8 software (Akoya Biosciences) was used for spectral deconvolution. Deconvoluted multispectral images were subsequently fused in HALO (Indica Labs).

### Figure preparation

Schematics for Figs. 1, 2 and 3 were prepared in BioRender.

### Supplementary Information

Supplementary Material 1.

## Data Availability

FAST5 and BAM files are available from ENA under accession PRJEB62796 (https://www.ebi.ac.uk/ena/browser/home). The different NRG1-VII isoform sequences and expected proteins are available at NCBI under submission numbers OQ272754-OQ272776 (https://www.ncbi.nlm.nih.gov/). The processed data and scripts used in this study are available at https://github.com/wellslab/NRG1-VII_Amplicons. IsoLamp is available on github at https://github.com/ClarkLaboratory/IsoLamp.
