# Supplementary material for: Discovery of NRG1-VII: the myeloid-derived class of NRG1
Source: BMC Genomics. 2024 Aug 29;25:814. doi: 10.1186/s12864-024-10723-2 (PMC11360300; doi:10.1186/s12864-024-10723-2)
Supplement: Supplementary file 1 — Supplementary Material 1. [file 12864_2024_10723_MOESM1_ESM.pdf]

Supplementary data

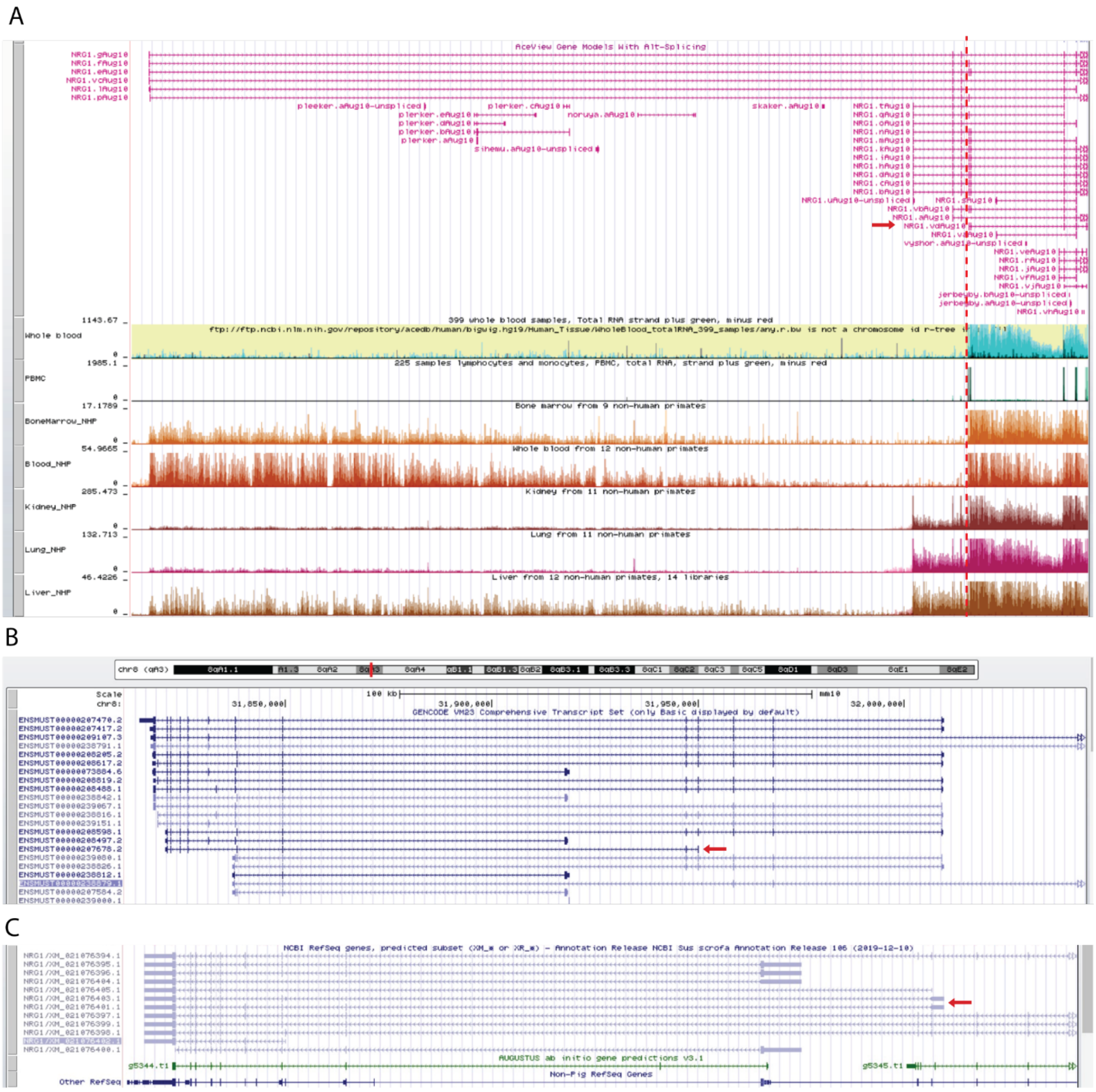

Supplementary Figure 1. NRG1-VII TSS shows expression conservation in primate and non-primate mammals. A) NRG1 expression profiles of human and non-human primates showing that TSS VII is conserved in bone marrow and whole blood **B)** Curated transcript in *Mus musculus* databases showing conservation of TSS VII in blood cells. **C)** Predicted transcript in *Sus scrofa* based ETSs derived from dendritic cells and other myeloid progenitor samples. Screenshot from https://www.ncbi.nlm.nih.gov1. *Accessed on 15/12/2022.*

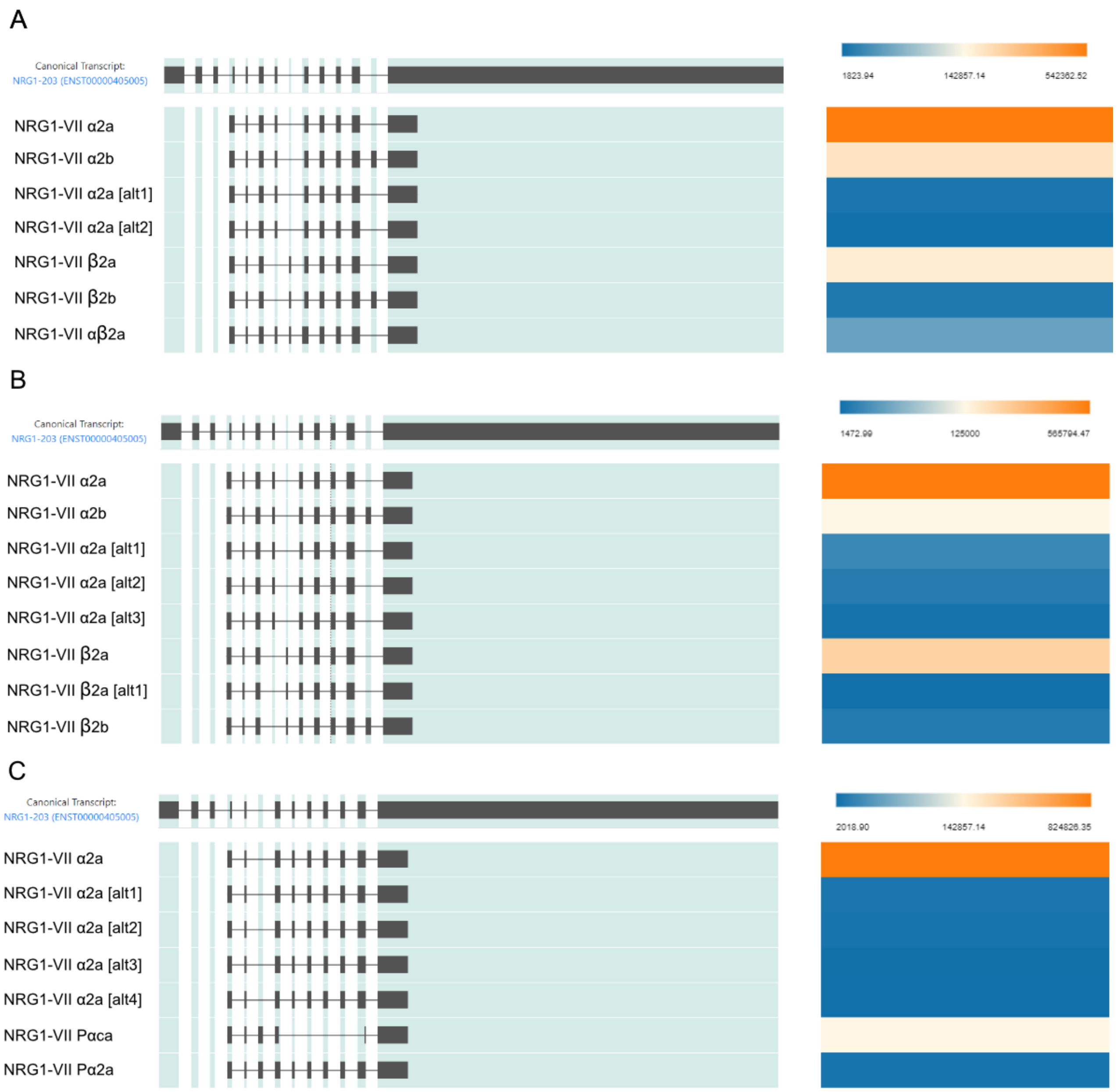

Supplementary Figure 2. NRG1 long amplicon isoforms found through IsoLamp. Each panel shows the IsoVis (Ref) view for NRG1 amplicons in a different sample. The initial row shows the canonical isoform structure chosen by Isomix. The rest show the resulting isoforms found by the IsoLamp analysis pipeline. Isoforms that include the term “[alt]” show isoforms that are only different to the main isoform in one of the splicing junctions by a few bases. The heat maps on the right-hand side describe relative abundance of each transcript in that sample in nTPM. Panels correspond to **A)** iPSC derived myeloid progenitors; **B)** iPSC derived macrophages (iMACs); **C)** Monocytes.

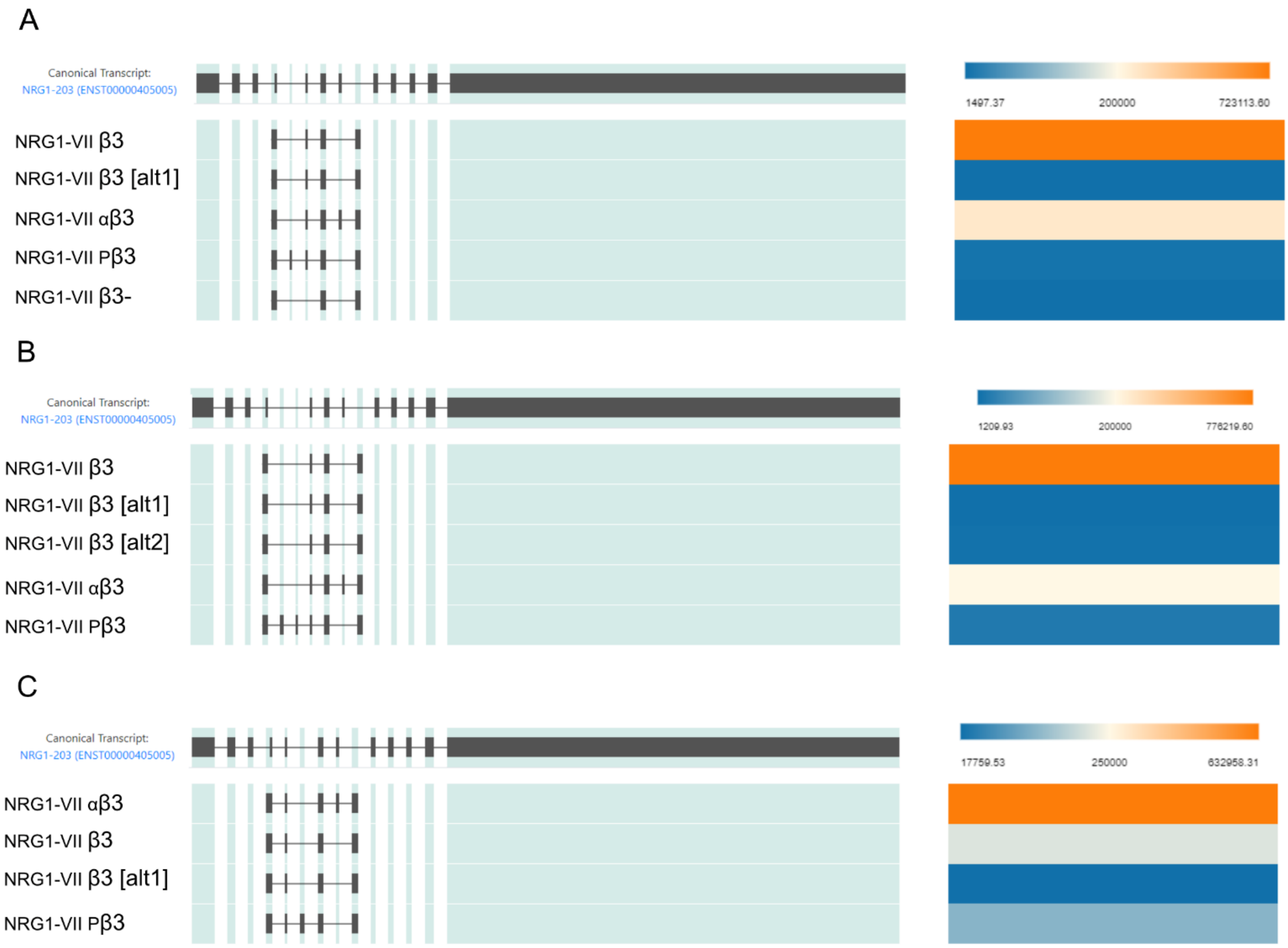

Supplementary Figure 3. NRG1 short amplicon isoforms found through IsoLamp. Each panel shows the IsoVis (Ref) view for NRG1 amplicons in a different sample. The initial row shows the canonical isoform structure chosen by Isomix. The rest show the resulting isoforms found by the IsoLamp analysis pipeline. Isoforms that include the term "[alt]" show isoforms that are only different to the main isoform in one of the splicing junctions by a few bases. The heat maps on the right-hand side describe relative abundance of each transcript in that sample in nTPM. Panels correspond to **A)** iPSC derived myeloid progenitors; **B)** iPSC derived macrophages (iMACs); **C)** Monocytes. Different poison exons were detected for isoform NRG1-VII Pβ3 in each sample type, therefore further validation of these exons and isoform(s) is needed.

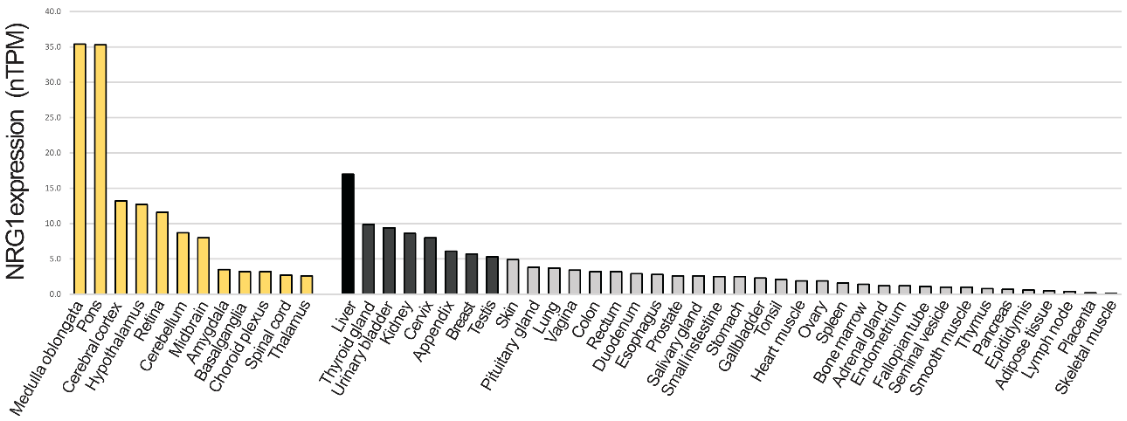

Supplementary Figure 4. NRG1 expression levels in macrophages present in a wide variety of human tissues. Quantification of NRG1 mRNA expression in macrophages present different human tissues according to The Human Protein Atlas (Karlsson et al., 2021). The y axis shows the number of transcripts per million. Yellow bars = neural tissue; Grey scale bars = levels of expression in all other tissues.

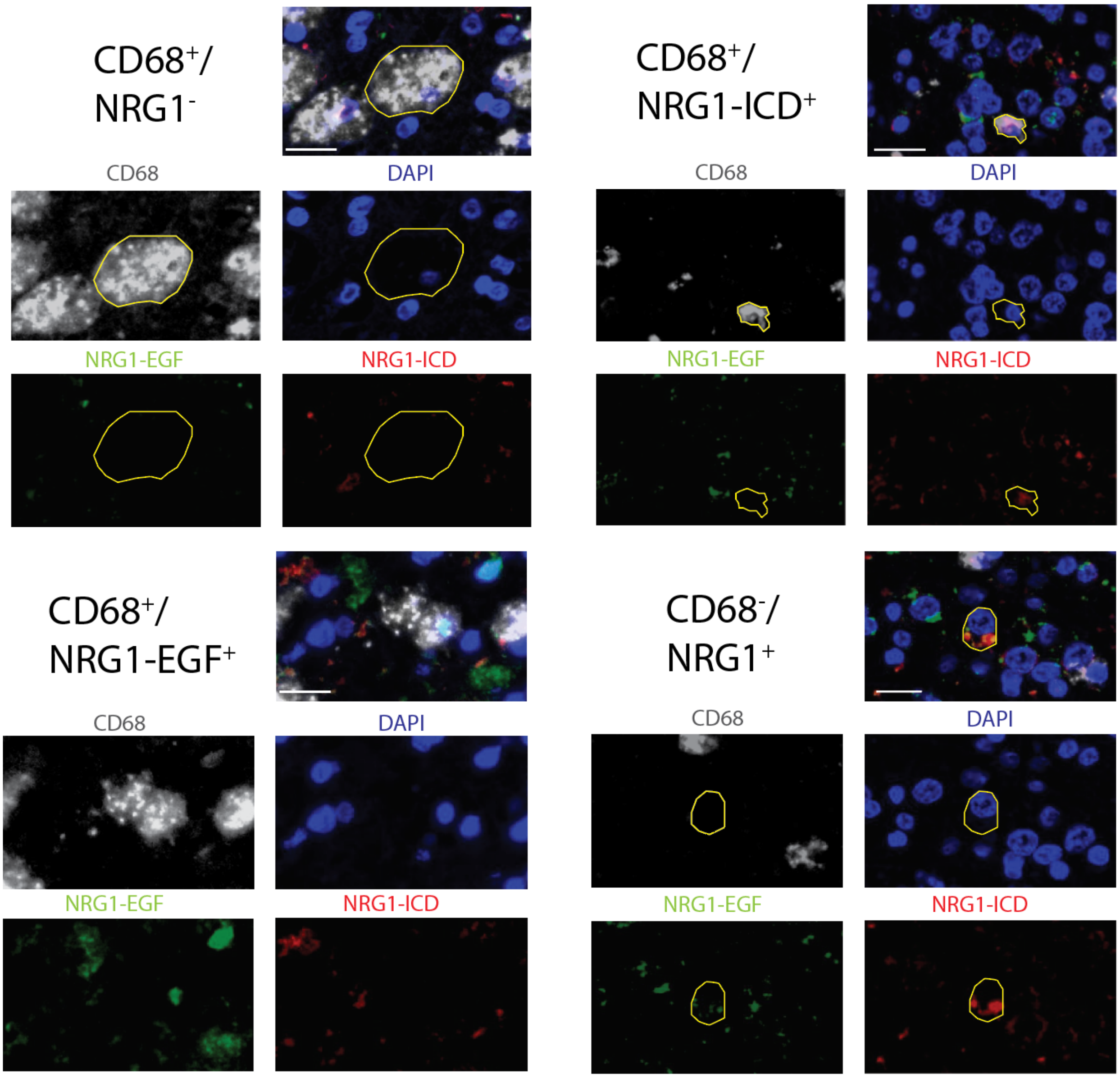

Supplementary Figure 5. Panel of marker combinations present in GBM samples. $CD68^+$ cells are diverse in their NRG1 expression patterns. Panels show $CD68^+$ cells that can be $NRG1^-$, or positive for either of the NRG1 antibodies independently. $CD68^-$ cells were also observed to be positive for presence NRG1. Scale bars = 20 µm.
